# Gaps in communication between different staff groups and older adult patients foster unnecessary antibiotic prescribing for urinary tract infections in hospitals: a qualitative translation approach

**DOI:** 10.1186/s13756-019-0587-2

**Published:** 2019-08-05

**Authors:** Paula M. Saukko, Beryl A. Oppenheim, Mike Cooper, Emily K. Rousham

**Affiliations:** 1 School of Social Sciences, Loughborough University, Loughborough, LE11 3TU UK; 2 Infection Prevention Team, New Cross Hospital, Royal Wolverhampton NHS Foundation Trust, Wolverhampton, WV10 0QP UK; 3 NIHR Surgical Reconstruction and Microbiology Research Centre, University Hospitals of Birmingham NHS Foundation Trust, Birmingham, B15 2GW UK; 4 School of Sport, Exercise and Health Sciences, Loughborough University, Loughborough, LE11 3TU UK

**Keywords:** Antibiotic prescribing, Antimicrobial resistance, Urinary tract infection, Diagnosis, Qualitative research

## Abstract

**Background:**

Studies have reported large scale overprescribing of antibiotics for urinary tract infection (UTI) in hospitalised older adults. Older adults often have asymptomatic bacteriuria, and clinicians have been found to diagnose UTIs inappropriately based on vague symptoms and positive urinalysis and microbiology. However, the joined perspectives of different staff groups and older adult patients on UTI diagnosis have not been investigated.

**Methods:**

Thematic analysis of qualitative interviews with healthcare staff (*n* = 27) and older adult patients (*n* = 14) in two UK hospitals.

**Results:**

Interviews featured a recurrent theme of discrepant understandings and gaps in communication or translation between different social groups in three key forms: First, between clinicians and older adult patients about symptom recognition. Second, between nurses and doctors about the use and reliability of point-of-care urinary dipsticks. Third, between nurses, patients, microbiologists and doctors about collection of urine specimens, contamination of the specimens and interpretation of mixed growth laboratory results. The three gaps in communication could all foster inappropriate diagnosis and antibiotic prescribing.

**Conclusion:**

Interventions to improve diagnosis and prescribing for UTIs in older adults typically focus on educating clinicians. Drawing on the sociological concept of translation and interviews with staff and patients our findings suggest that inappropriate diagnosis and antibiotic prescribing in hospitals can be fuelled by gaps in communication or translation between different staff groups and older adult patients, using different languages and technologies or interpreting them differently. We suggest that interventions in this area may be improved by also addressing discrepant understandings and communication about symptoms, urinary dipsticks and the process of urinalysis.

**Electronic supplementary material:**

The online version of this article (10.1186/s13756-019-0587-2) contains supplementary material, which is available to authorized users.

## Introduction

Urinary tract infections (UTIs) are the second biggest source of antibiotic prescribing in the UK and a major contributor to antimicrobial resistance (AMR) [1]. Diagnosing UTIs in older adults is challenging, as they frequently have asymptomatic bacteriuria (ASB), which is often inappropriately treated with antibiotics [2]. UK clinical guidance recommends that UTI diagnosis in older adults should be primarily based on symptoms^1^; point-of-care urinary dipsticks are not recommended, and bacterial culture is not a reliable diagnostic test but is recommended to confirm diagnosis and to determine sensitivities to antimicrobials [1, 3].

Research in the UK [4], US [5] and Korean [6] hospitals has reported that between 30 and 40% of antibiotic prescribing for UTIs are cases of ASB. Interviews with junior doctors found that antibiotic overprescribing was driven by overreliance on laboratory results, fear of poor outcomes, perceived pressure from peers and patients, and difficulties in interpreting symptoms [7]. Qualitative study on hospital clinicians found that misdiagnosis of UTIs was driven by unreflective use of urinary dipsticks [8], and our parallel case series review of patient records also found that 54% of older adults had a dipstick recorded [9]. A survey of junior doctors reported that a third were unaware of antibiotics not being indicated for ASB, but 46% of doctors, who were aware of recommendations, reported still prescribing for ASB [6]. Qualitative interview studies in care homes in UK and Canada have observed that nurses often diagnose UTIs based on vague symptoms, such as foul-smelling urine, and GPs prescribe antibiotics over the phone without seeing the patient [10, 11].

A recent systematic review of qualitative research on antibiotic prescribing in hospitals observed that doctors focused on the individual patient and considered the risk of AMR for them small, driving suboptimal prescribing [12, 13]. Nurses have been found to advocate for prescribing [14], and interviews with clinicians discovered a ‘prescribing etiquette,’ whereby prescribing decisions were made to appease other staff and patients [15, 16]. Junior doctors have been found to feel unsupported in their antibiotic prescribing decisions [17]. Interviews have also identified barriers to communication between microbiology laboratories and clinical units [18].

These studies highlight the importance of the interplay between different staff groups, patients and clinical domains in antibiotic prescribing. However, the joined perspectives of different staff groups and older adult patients on diagnosing and prescribing for UTIs in hospitals have not been studied; we did not identify any studies on older adult patients themselves. Our study offers the first investigation of the differing perspectives of nurses, doctors, older adult patients and microbiologists on the UTI diagnostic pathway, highlighting how these differences contribute to unnecessary antibiotic prescribing for UTIs.

The study takes a conceptual lead from the notion of translation, as discussed in sociology of science [19]. Different groups of people, such as lay people and scientific experts as well as patients and different healthcare staff, have their own specific languages and routines, mediated by technologies, such as urinary dipsticks and electronic test results. Collaboration across these groups requires translation, as when a nurse translates a patient’s embodied experience of illness into clinical symptoms or when a urine specimen is translated into bacterial counts by microbiology. Our research suggests that key problems in diagnosis and prescribing for UTIs in older adults in hospitals relate to gaps in communication or translation between different staff groups and patients in specific instances along the UTI diagnostic pathway.

## Methods

The study was conducted between December 2016 and September 2017 in one large and one community hospital, to capture different contexts, in the UK Midlands. Sampling was purposeful; since previous research had focused on doctors, we sought to capture the joined perspectives of nurses, doctors, older adult patients and microbiologists. After receiving NHS Health Research Authority approval for the study (IRAS 202255), posters were displayed in wards and information about the study was circulated via email in relevant hospital newsletters. A research nurse followed up with regular visits to wards and distributed invitations to participate and information sheets for (i) healthcare staff involved in diagnosing UTIs in older adults and (ii) older adult (≥70 years) patients, who had been diagnosed with a UTI during the current hospital stay and were cognitively capable of giving written informed consent. Staff and patients who expressed an interest in participating were contacted by an experienced qualitative researcher, who arranged for an interview.

We recruited a total of 41 participants, comprising 13 nurses, 3 healthcare assistants (HCAs), 9 doctors, 14 patients, and 2 microbiologists. Recruitment was stopped when saturation was reached for the key themes identified from the interconnected perspectives of healthcare staff and patients. Most of the doctors [7] were junior doctors, who are mostly in charge of initial UTI diagnosis; they were recruited from both acute and subacute wards. The nurses and patients were recruited from subacute wards, including older adults, rehabilitation, orthopaedics and stroke. The average age of the patients was 81 years (range 71–89 years).

The study was introduced in terms of finding out how UTIs are diagnosed and treated in older adults. The healthcare staff were asked about their job role and their role in and experiences of diagnosis and treatment of UTIs in older adults, prompting for symptom recognition, use and interpretation of diagnostic tests, collection of urine, antibiotic prescribing and any concerns. Patients were asked about their experiences of being diagnosed and treated for UTIs, prompting for symptoms, interpretation of tests, treatment, previous UTIs and overall health. The interviews were transcribed verbatim and analysed for themes, using the constant comparative method [20], facilitated by NVivo 11 qualitative software. The initial coding was done by PS, a sample of transcripts was read by other team members, and the final coding scheme was developed through building consensus via conversation between members of the research team. The coding scheme with illustrative quotes is provided as Additional file 1.

We identified a recurrent overarching theme of gaps in communication in the interviews with three forms: (i) between clinicians and older adult patients about symptoms, (ii) between nurses and doctors about interpretation of urinary dipsticks and (iii) between nurses, doctors and microbiologists about collection of urine specimens and interpretation of bacterial cultures. In what follows we will discuss each theme in detail.

## Results

### Communication about symptoms

The interviews highlighted gaps in communication between clinicians and older adult patients about UTI symptoms.

Clinicians often focused on non-specific signs of UTI that they could observe without discussing with the patient. The most common of such signs were confusion, general unwellness or a sudden deterioration or being *off their baseline* (P22, nurse), for example:

Most of them are coming in with symptoms of confusion … Also, they are coming in with lethargy … so in general the patient is kind of deteriorating (P2, nurse).

A new onset or worsening confusion is one of the signs and symptoms of a UTI, but it is non-specific, being associated with diverse morbidities and frailty. Particularly nurses, but also doctors, frequently mentioned urine with a foul odour, which is not an indication of UTI but can be a sign of dehydration:

Usually, the rehab assistants will tell us, oh we found this lady, and the urine’s really concentrated, or it’s offensive smelling and, it’s by observation isn’t it, by looking and smelling. And the other one is if we find them a little confused, or the patient might say, oh it really burns when I have a wee (P14, nurse)

The excerpt above further illustrates that clinicians often mentioned dysuria, which is a specific symptom for UTIs, however, it was typically not the first symptom brought up. Clinicians noted that dysuria was often difficult to diagnose in older adults due to poor health or cognitive impairment, so they focused on signs that could be observed without communicating with the patient:

The traditional symptoms that a patient might describe to you would be burning, stinging … But patients aren't always able to describe those symptoms, so it might be reports from nurses, that the urine is smelly (P3, doctor).

Older adult patients typically first brought up dysuria i.e. pain when describing symptoms. Some described the intensity of pain, such as *trembling, trying to control the pain* (P33). However, other patients stated that they had felt only minor discomfort or *tiny bit of smarting* that they did not associate with *cystitis* (P18) or described that they did not have symptoms of dysuria when diagnosed with a UTI:

**Did you notice any pain whilst —?** No, not at all. My water wasn’t burning or anything like that, it was just slightly cloudy apparently (P6, patient).

The excerpt indicates that the clinician had focused on an inaccurate sign of UTI and most likely overlooked the patient’s lack of symptoms of dysuria. The problem here is a focus on inaccurate signs, but most importantly patient’s lucid account of lack of symptoms also suggest that clinicians do not necessarily adequately communicate with the patients and elicit symptoms of dysuria or lack thereof.

However, a few patients had been too ill to make sense of or recall the events at the time of diagnosis, indicating it had likely been difficult for them to communicate with clinicians:

It seems I got this infection, and I finished up in here in this hospital. **Were you poorly?** I was. … **Did somebody call an ambulance, do you remember?** I don’t know what happened really (P41, patient).

Overall, there was a discrepancy between clinicians’ and older adults’ foci when describing signs and symptoms of UTI. Clinicians typically focused on non-specific or inaccurate signs and symptoms of UTI they could observe without communicating with the patients. Older adult patients associated UTI first and foremost with the specific symptom of pain when urinating and also sometimes described being diagnosed without dysuria, indicating in the interviews they were mildly surprised with being diagnosed without symptoms. These findings corroborate previous research that clinicians focus on vague signs and symptoms of UTI in older adults [7, 10, 11]. However, more specifically the interviews with older adult patients indicate gaps in communication between clinicians and patients about symptoms, clinicians not necessarily effectively eliciting symptoms of dysuria or lack thereof. Nevertheless, the clinicians’ and a few patients’ interviews also indicate that sometimes communication with older adult patients was challenging due to sudden or long-term ill health and confusion.

### Interpreting urinary dipsticks

There was also a discrepancy between how nurses and doctors interpreted point-of-care urinary dipsticks, which indicate the presence of nitrates and leucocytes, by-products of bacteria, in urine.

Nurses typically reported that *urine dip* (P8, nurse) was performed when a patient showed signs or symptoms of UTI or as part of admissions routines. Most nurses considered dipsticks as part of nursing routines and considered them reliable:

**What’s your perspective on the reliability of the dipstick, is it trustworthy?** I would hope so, yeah. I’ve been nursing for a while, and no-one’s ever said that it’s not (P22, nurse)

Doctors often doubted the reliability of dipsticks. Some had been instructed that *the elderly patients can naturally have leucocytes* (P16), others had concluded this based on their clinical experience, observing that *mild white cells is quite common in older people* (P26) or that positive dipstick results were often not corroborated by positive bacterial cultures (P5). Many criticised the use of dipsticks:

I think we put a lot of reliance on the urine dip, and … if you read about the sensitivity of urine analysis, most older adults … can have white cells in their urine dip, which does not correlate with having a urinary tract infection (P25, doctor)

Yet, doctors stated that positive dipstick results, which were immediately available, often tipped the balance toward prescribing antibiotics for a UTI, when patients presented as *not well clinically* (P24, doctor):

Very commonly a patient will be confused, so the nurses, by default, will dip them, they will be positive for nitrates, and then by default the doctor will prescribe antibiotics (P16, doctor).

Older adult patients considered positive dipstick results (and bacterial cultures) to confirm they had a UTI:

They must have had a look at my water – I had to give a water – **So they tested your water in the hospital?** And they said I’d got a urine infection (P31, patient)

Nurses and patients, thus, considered positive dipstick results to indicate a UTI, whereas doctors doubted the reliability of dipsticks. However, the dipstick results made available by the nurses guided doctors’ prescribing decisions. These findings indicate widespread inappropriate use of dipsticks to diagnose UTIs in older adults, which was also found in our parallel case series review [9] and that was not sufficiently addressed by hospital policies or education of clinicians, particularly nurses. However, the findings also suggest poor communication between nurses, doctors and patients on use and interpretation of urinary dipsticks, which fostered inappropriate diagnosis and antibiotic prescribing.

### Communication between clinicians and the laboratory

The third gap in communication identified in the interviews related to potential contamination of urine specimens and occurred between nurses collecting urine specimens, microbiologists processing urine cultures and doctors interpreting laboratory results.

Nurses and HCAs stated that it was usually not possible to obtain a midstream urine specimen from older adult patients due to illness, immobility and/or incontinence. Urine was collected, typically with a syringe, from containers, bedpans, convenes, continence pads and catheter bags, nurses mentioned using Newcastle urine collection pads, but their use was discontinued due to high cost:

They’re incontinent. … a lot of them will have their bowels open at the same time – I know people say MSU [Midstream Specimen of Urine], but it’s not an MSU we take, it’s the clean catch. We used to use the Newcastle pads, but we haven’t had the Newcastle pads for a long, long time – … you know, they walk to the toilet, pop a bed pan there, and we can catch it then (P20, nurse).

Doctors were aware of urine specimens not being collected midstream:

The traditional sort of midstream urine, I'm sure, happens very rarely in hospital. I think if we can get any urine, then we're just happy that we've got something to test (P25, doctor).

Some patients described specimens being taken from bedpans due to immobility:

I gave them a sample … I had to use a bed pan, I couldn’t. I can't walk you see, since I’ve had the stroke (P35, patient).

Other patients indicated that they had not been instructed on how to collect a midstream specimen, suggesting another problem in communication between clinicians and older adult patients:

**How did they ask you to take the urine? Did you have to pee in a pot?** Yes, but they brought their own thing to make sure everything is clean. **And did they tell you to pass it for a little bit and then take it in the middle? Or did they give instructions?** Nothing, no. Just they say, go and do something, and then they go and have a look at that (P37, patient)

Microbiologists noted that a significant portion of bacterial cultures from older adults were mixed growth, which they presumed was due to specimens being contaminated:

The elderly patients, it’s kind of an assumption that we make is they’re not as mobile, if they grow a mixed culture it could be just to do with general cleanliness … if like two or three or four or more things grow then it’s unlikely to be a true urine infection, but it could be to do with the condition of the patient and how well that sample is collected (P7, microbiologist).

Microbiologists also stated they were *not sure* mixed growth results were *fully understood by the clinicians* (P11). Indeed, many junior doctors were uncertain how to interpret mixed growth results and could interpret them requiring broad spectrum antibiotics (P27); most doctors were unsure whether the results indicated treatment or not:

[Interpreting laboratory results is difficult when] they’ve got high white cell counts in urine but no significant growth. I don’t actually know what that means or whether you should treat or not. **How about mixed growth?** Mixed growth is another one, because with mixed growth you don’t normally get sensitivity either (P28, doctor)

Research in hospitals and care homes has reported that urine specimens from older adults are frequently not collected midstream and that patients are often not instructed how to collect a midstream urine specimen [5, 11]. What our findings add is to show that doctors, nurses and microbiologists were aware of problems in urine collection but not of their downstream implications. Our findings highlight that gaps in communication or translation between different staff groups, patients and clinical domains along the UTI diagnostic pathway in hospitals could lead to inappropriate diagnosis of UTIs in older adults and unnecessary antibiotic prescribing when junior doctors were uncertain whether to prescribe for large volumes of mixed growth results originating from contaminated specimens.

## Discussion

Interventions to improve diagnosis and prescribing for UTIs in older adults in hospitals and care homes have focused on educating individual clinicians about clinical guidance and evidence and have incorporated educational sessions, pocket cards, posters, algorithms and feedback [21–25]. Most of these interventions have achieved reductions in unnecessary antibiotic prescribing at least in the short term [21, 23, 24], and a recent Cochrane Review on interventions to improve antibiotic prescribing in hospitals found most of them effective [26]. It has been suggested that what is needed to drive further improvements is not further trials on similar interventions but conceptually informed understanding of the social and behavioural elements and processes influencing prescribing in different contexts [27].

Our findings suggest that there is a knowledge or education deficit among clinicians about correct signs and symptoms of UTI and use of urinary dipsticks in diagnosing older adults. Most importantly, however, our findings suggest that there are significant discrepancies between the perspectives on UTI diagnosis between different staff groups and older adult patients, which results in miscommunication that fosters unnecessary antibiotic prescribing. Thus, addressing these gaps in communication between clinicians and patients and between different staff groups and clinical domains could importantly enhance antimicrobial stewardship efforts and interventions in the complex context of hospitals.

The importance of social interactions, shared understandings and work practices as well as understanding how different actors and elements play together in hospitals has been acknowledged in psychological, sociological and healthcare systems theories on behaviour change and implementation [28–30]. Our research has been informed by the sociological concept of translation [19], which highlights how different professional and lay groups use different languages, mediated by routines and technologies, such as urinary dipsticks being an integral part of nursing routines. This concept illustrates how messages across the UTI diagnostic pathway often get lost when information is translated between social groups, who approach the situation from a different perspective.

The first key problem in translation and communication we identified occurred between clinicians and older adult patients about UTI symptom recognition. Clinicians typically focused on vague, observable signs of UTI, and based on patient accounts clinicians could overlook patients’ lack of symptoms of dysuria. Diagnosis of UTIs in older adult should primarily be based on symptoms [1], and research, including our parallel case series review, has consistently found that UTI diagnosis is often not based on appropriate symptoms [5–7, 9]. Our findings suggest that efforts to improve antibiotic prescribing for UTIs could be importantly enhanced by developing advice for clinicians on how to not only recognise but also communicate with older adult patients about symptoms. Communication is particularly important for discerning the main specific symptom of UTI, pain when urinating, which is most effectively identified by eliciting patients’ subjective experiences rather than observation.

The second gap in communication identified was between nurses, doctors and patients, who interpreted urinary dipsticks differently. Our parallel case series review found that 54% of older adult patients had a urinary dipstick recorded [9], and qualitative and quantitative research in care homes and hospitals have reported clinicians to overrely on positive urinalysis in diagnosing UTIs, fuelling antibiotic prescribing for asymptomatic bacteriuria [4–7, 9]. These findings underline the importance of raising clinicians’ awareness of the unreliability of dipsticks in diagnosing older adults. What our study adds to this picture is the observation that nurses routinely used and relied on dipsticks, whereas doctors typically doubted their reliability but the results presented still influenced their antibiotic prescribing, and older adult patients took it for granted that “testing the water” confirmed a UTI. Thus, poor communication between different staff groups and older adult patients about the use and reliability of dipsticks fomented an ingrained inappropriate routine of using them to inform diagnosis and prescribing for UTIs in older adults. Creating ways to improve communication between diverse staff and patients about diagnostic tests is particularly important against findings that antibiotic prescribing decisions are frequently informed by a “prescribing etiquette” whereby decisions are made to appease colleagues, patients and families [16].

The third gap in communication found was between nurses, doctors, older adult patients and microbiologists related to potentially contaminated urine specimens. Research has reported urine specimens frequently being collected inappropriately from older adults [5, 11]. Our findings corroborate this and healthcare staff was aware of the problem. However, healthcare staff was not aware of the downstream implications of problems in urine collection. Our case series review showed that 38% of urine cultures for older adults were mixed growth [9]. Our qualitative findings indicated that junior doctors were often uncertain of how to interpret mixed growth results and could interpret them as requiring broad spectrum antibiotics. Previous research has found barriers in communication between clinical units and microbiology laboratories. Our findings suggest that promoting shared understanding of the process of urinalysis between diverse staff, patients and clinical domains could improve practices and reduce unnecessary antibiotics.

The strength of our study is that we examined the joined perspectives of nurses, doctors, older adult patients and microbiologists, highlighting problems in communication or translation along the UTI diagnostic pathway in hospitals. The limitation of the study is that we focused on just two hospital trusts and, in principle, aspects of our findings can reflect idiosyncrasies in the two trusts at a particular time. However, international quantitative and qualitative literature has reported inappropriate diagnosis of UTIs vis a vis symptom recognition and use of dipsticks in hospitals [5–7], so our findings, focusing on the role of communication or translation in these processes, are likely to resonate with or be transferable to similar contexts. Our parallel case series review of patient records [9] corroborated the qualitative observations on UTI diagnosis meaning they should broadly reflect practices in these local contexts. Further, whilst our overall sample was robust for a qualitative study (*n* = 41), the subgroups of staff groups and patients were small. For this reason our analysis focused on themes that repeated across the subgroups, however, we were not able to explore diversity within them, for example, whether junior and senior doctors perspectives were different. We also excluded older adult patients with significant cognitive impairment who could not give informed consent, and it is useful to bear this in mind when interpreting the findings.

## Conclusions

Interventions to improve diagnosis and antibiotic prescribing for UTIs in older adults have typically focused on educating clinicians. Drawing on the concept of translation and interviews with different staff groups and older adult patients our findings suggest that inappropriate diagnosis and antibiotic prescribing in hospitals can be fuelled by gaps in communication or translation between different staff groups and patients, using different languages and technologies or interpreting them differently. We suggest that interventions in this area may be improved by also addressing discrepant understandings and communication about symptoms, urinary dipsticks and the process of urinalysis.

## Additional file

Additional file 1: Coding scheme with illustrative quotes. (DOCX 32 kb)

## Data Availability

Under the conditions of the Health Research Authority approval the data are not publicly available for sharing. Requests for data sharing can be made to the study sponsor.

## Abbreviations

AMR: Antimicrobial resistance
ASB: Asymptomatic bacteriuria
HCA: Healthcare assistant
MSU: Midstream specimen of urine
UTI: Urinary tract infection

## Footnotes

1 Signs and symptoms include the presence of two or more of the following: dysuria, urgency, frequency, urinary incontinence, shaking, chills, flank or suprapubic pain, or new onset of or worsening of pre-existing confusion /agitation [1].
