# Supplementary material for: Gaps in communication between different staff groups and older adult patients foster unnecessary antibiotic prescribing for urinary tract infections in hospitals: a qualitative translation approach
Source: Antimicrob Resist Infect Control. 2019 Aug 5;8:130. doi: 10.1186/s13756-019-0587-2 (PMC6683464; doi:10.1186/s13756-019-0587-2)
Supplement: Supplementary file 1 — Coding scheme with illustrative quotes. (DOCX 32 kb) [file 13756_2019_587_MOESM1_ESM.docx]

Gaps in communication between different staff groups and older adult patients fostering unnecessary antibiotic prescribing urinary tract infections in hospitals: A qualitative translation approach to improvement

Coding scheme with illustrative quotes

Paula Saukko

2019

| Theme | Subtheme | Illustrative quotes |
| --- | --- | --- |
| Gaps in communication between clinicians and patients about  signs and symptoms of UTIs | Clinicians’ focus on observable, non-specific  or inaccurate signs and symptoms; dysuria often mentioned as an afterthought or considered difficult to diagnose | ‘Most of them are coming in with symptoms of confusion ... Also, they are coming in with lethargy … so in general the patient is kind of deteriorating’ (P2, nurse).  Usually, the rehab assistants will tell us, oh we found this lady, and the urine’s really concentrated, or it’s offensive smelling and, it’s by observation isn’t it, by looking and smelling. And the other one is if we find them a little confused, or the patient might say, oh it really burns when I have a wee (P14, nurse)  We tend to have confusion, disorientated. They might be quite sleepy, sometimes thirsty, irritated down below, a new incontinence, these are the symptoms we tend to see. (**Is it**) **difficult to diagnose symptoms in the older adult?** It is more so in the acute and in the elderly because they don’t usually come in with one symptom. Their confusion can be – although it’s a predominant factor, it can be masking other things that’s going on as well (P9, nurse).  The traditional symptoms that a patient might describe to you would be burning, stinging … But patients aren't always able to describe those symptoms, so it might be reports from nurses, that the urine is smelly (P3, doctor)  If the patient’s a bit more confused or agitated than their baseline, or if they’re wandering at night, or if they’re complaining of some sort of dysuria or sort of abdominal pain. And occasionally it will be nursing staff will just think that their urine smells a bit offensive or looks cloudy (P27, doctor)  With our patient population I’d be looking for delirium, confusion, a fall, off their feet, reduced mobility. ... we have more dementia patients, that tend to be more confused – not all the time ... Sometimes they can't really accurately describe what’s happening to them. So, you have to look for the signs. Rather than ask. (P28, doctor) |
|  | Patient experiences of lack of symptoms, description of clear symptoms or confusion / inability to recall events | **Did you notice any pain whilst —?** No, not at all. My water wasn’t burning or anything like that, it was just slightly cloudy apparently (P6)  Well, my legs gave way and then they rushed me in ... **Did you have pain like in your lower stomach or anything?** No. **No, or like did you have trouble passing urine?** No (P31).  **Did you have pain or symptoms or—?** No, I don’t have pain … but I was very constipated …and after I’d been for the toilet, there was just a tiny little bit of smarting … I know what cystitis is because I’ve had that three or four times. It wasn’t like that, just smarted a little tiny bit and then it went (P18)  I was really in pain. And my leg kept trembling, trying to control the pain. I said I feel I need to go pee and nobody is taking any notice. So, he went and talked to someone, and they realised I had an infection (P33).  **Did you have pain?** Yes. **Like in your —** Yes, I had pain passing water. I still do a little bit but not so bad (P32).  **So what are the symptoms you get when the UTI starts cropping up?** Short and simple, pain. Wife: *He gets pain, but I can see that he’s just not with it. He’s totally out of it. It all starts with a tremor of lips and mouth … And he doesn’t understand to do what you want him to* (P38).  It seems I got this infection, and I finished up in here in this hospital. **Were you poorly?** I was. … **Did somebody call an ambulance, do you remember?** I don’t know what happened really (P41) |
| Gaps in communication between nurses, doctors and patients about  urinary dipsticks and prescribing | Nurses’ reliance on  urinary dipsticks | **What’s your perspective on the reliability of the dipstick, is it trustworthy?** I would hope so, yeah. I’ve been nursing for a while, and no-one’s ever said that it’s not (P22, nurse)  **Do you find [the dipstick] reliable?** How do I find it – it’s a bit strange, isn’t it. No, I think we probably do trust them because that’s the simple diagnostic tool we’ve got here … that’s all we really have to go on, apart from your own observations of colour and any odour (P15, nurse)  [According to new instructions we have to] wait for it [the sample] come back on the system as a diagnosed urinary tract infection before they treat it. .. **So, okay, well why do you still do the dipstick?** You know, okay— We’re told really. Nurse training, if it looks off, dip it, see whether it’s got any protein or leucocytes in it or anything that looks like it might be a sign of anything growing. Yeah, just kind of like a first sort of quick— Habit, I suppose (P8, nurse). |
|  | Doctors’ doubts about urinary  dipsticks | I think we put a lot of reliance on the urine dip, and … if you read about the sensitivity of urine analysis, most older adults … can have white cells in their urine dip, which does not correlate with having a urinary tract infection (P25, doctor)  So how often does the dipstick and the urine culture differ? A lot. A lot. Yes. So, why? Because, in the elderly you get what's called asymptomatic bacteria, which is where you can get white cells in the urine but not actually associated with an infection. So there might be no bugs there. So, if you're just treating purely on the dipstick, then you end up treating a lot more (P3, doctor)  How do you establish, rule out the UTI? … I ask for a urine specimen, so we’ll get it dipped, but I don’t really use that to be honest. If it’s negative then it’s negative, but I’m quite aware that there’s quite a lot of false positives and false negatives with the urine dip, so we’ll always ask for it to be sent off (P27, doctor) |
|  | Clinicians’ accounts of antibiotics prescribed based on dipstick results | Very commonly a patient will be confused, so the nurses, by default, will dip them, they will be positive for nitrates, and then by default the doctor will prescribe antibiotics (P16, doctor)  So once the patient comes up to acute medicine we do a urine dip and if it’s positive for nitrites, leukocytes, we generally ask them to send it to the lab for culture and see if it’s growing anything. If there are any signs of sepsis or white cell count is too high, CRP is high, patient is not clinically well, we start the patient on antibiotics anyway (P24, doctor)  **How would you describe your prescribing practise?** If we have reliable evidence on a dip for a urine infection, so leukocytes and nitrites, I will often prescribe the commonest antibiotic for urine infection, without waiting for the culture result (P26, doctor) |
|  | Patients’ interpretation of  diagnostic tests as confirming UTI | They must have had a look at my water – I had to give a water – **So they tested your water in the hospital?** And they said I’d got a urine infection (P31, patient)  I think what’s happening is I’m going back to the hospital to see a doctor, take a water sample to see if they’ve done the job or whether there’s still something there (P29, patient)  Every so often, when the district nurses come, they use a dipstick because I say to them, "Look, please test it," and they do that. Then if there's any query, any doubt about the result, they take a proper sample and get it sent to the lab (P40, patient). |
| Gaps in communication between clinicians and laboratory | Nurses’ description of non-midstream urine collection | They’re incontinent. … a lot of them will have their bowels open at the same time – I know people say MSU [midstream urine], but it’s not an MSU we take, it’s the clean catch. We used to use the Newcastle pads, but we haven’t had the Newcastle pads for a long, long time – … you know, they walk to the toilet, pop a bed pan there, and we can catch it then (P20, nurse)  If they’re quite cooperative, it’s really easy if they go to the toilet. But sometimes they’ve got pads on and, you know, it’s so difficult to try and get a urine sample. So sometimes it’s just a matter of kind of sitting them on a bedpan for five, ten minutes a couple of times a day just to see if you can get enough to just get a urine sample. **Okay, so do you go to the toilet with them to take the sample--?** Yeah, sometimes we kind of put like a bedpan on the toilet so we can collect the sample that way, yeah. **So do you try the midstream urine or is it too difficult?** Sometimes it’s too difficult (P21, HCA).  I mean if they’re continent you can just take them to the toilet. If they’ve got a catheter, you know, you can take it out the port. If they’re incontinent, we used to have these Newcastle pad things, but I think they were stopped a couple of years ago because of the expense really. So we just have to try our best to just take the patients to the toilets. And if it’s a man, maybe a convene, but if it’s a lady, just keep taking them and kind of running the tap really, try to get her to pass anything, but it is definitely a challenge with our females who are incontinent and they have like dementia and things like that (P22, nurse) |
|  | Patients’ description of urine collection as difficult or not being instructed | I gave them a sample … I had to use a bed pan, I couldn’t. I can't walk you see, since I’ve had the stroke (P35, patient)  **So do you mind me asking, how did they ask you to take the urine? Did you have to pee in a pot?** Yes, but they brought their own thing to make sure everything is clean. **Like a little pot with a syringe?** Yeah. **And did they tell you to pass it for a little bit and then take it in the middle? Or did they give instructions?** Nothing, no. Just they say, go and do something, and then they go and have a look at that (P37, patient).  **Did they take a urine sample?** Yeah. Oh, they said to me to do it in a little jar … **Did they tell you how to take it, like pass a little first —** Well, you fill one up. (Laughs) **They just say fill it up?** Yeah (P34, patient) |
|  | Doctors’ descriptions of urine collection as problematic | The traditional sort of midstream urine, I'm sure, happens very rarely in hospital. I think if we can get any urine, then we're just happy that we've got something to test (P25, doctor)  I think one of the most tricky patients to actually get urine from are the patients who have dementia or are just very confused and they’re incontinent. You can’t really capture the urine and you can’t really capture a good sample that’s, how do you say, it would grow something accurately and sterilely, being sterile. I think that’s a very challenging thing to do (P5, doctor). |
|  | Microbiologists’ description of mixed growth cultures as result of contamination | The elderly patients, it’s kind of an assumption that we make is they’re not as mobile, if they grow a mixed culture it could be just to do with general cleanliness … if like two or three or four or more things grow then it’s unlikely to be a true urine infection, but it could be to do with the condition of the patient and how well that sample is collected (P7, microbiologist)  So, the specimens we do get I suspect are just out of the bedpan and they take the bedpan into the universal container and clearly that’s going to be contaminated. **So do you think that the mixed growth tends to get treated?** It probably does get treated based on what they’ve already suggested from their clinical judgement and they probably view that as validation because it’s not a negative. … But it’s never been a validation, but it’s not a negative, it’s not a positive nor a negative, and I’m not sure that’s fully understood by the clinicians (P11, microbiologist). |
|  | Doctors’ uncertainty as to how to interpret mixed growth cultures | [The laboratory results are unclear when] they’ve got high white cell counts in urine but no significant growth. I don’t actually know what that means or whether you should treat or not. **How about mixed growth?** Mixed growth is another one, because with mixed growth you don’t normally get sensitivity either (P28, doctor)  If it’s heavy mixed growth it will say that. They’ll break it down sometimes, a certain number of white cell counts, white blood cells. The heavy mixed growth is my favourite, it’s tricky, how do you make sense of that? I think that’s probably for me one that I’m a little uncertain of. When I see heavy mixed growth I interpret that as meaning that they do have a UTI but they haven't been able to identify one specific organism. Therefore broad spectrum antibiotics I’d be choosing should be fine (P27, doctor)  One of the most ambiguous results is probably when it comes back with someone saying ‘heavy mixed growth’, because that means it can be anything, but there is obviously a bacteriuria (P16, doctor) |
